# Pelargonidin Modulates Keap1/Nrf2 Pathway Gene Expression and Ameliorates Citrinin-Induced Oxidative Stress in HepG2 Cells

**DOI:** 10.3389/fphar.2017.00868

**Published:** 2017-11-27

**Authors:** G. R. Sharath Babu, Tamatam Anand, N. Ilaiyaraja, Farhath Khanum, N. Gopalan

**Affiliations:** ^1^Biochemistry and Nano Sciences Division, Defence Food Research Laboratory, Mysore, India; ^2^Food Biotechnology Division, Defence Food Research Laboratory, Mysore, India

**Keywords:** citrinin, pelargonidin chloride, cytotoxicity, oxidative stress, mitochondrial membrane potential, Keap1/Nrf2 signaling pathway

## Abstract

Pelargonidin chloride (PC) is one of the major anthocyanin found in berries, radish and other natural foods. Many natural chemopreventive compounds have been shown to be potent inducers of phase II detoxification genes and its up-regulation is important for oxidative stress related disorders. In the present study, we investigated the effect of PC in ameliorating citrinin (CTN) induced cytotoxicity and oxidative stress. The cytotoxicity of CTN was evaluated by treating HepG2 (Human hepatocellular carcinoma) cells with CTN (0–150 μM) in a dose dependent manner for 24 h, and the IC_50_ was determined to be 96.16 μM. CTN increased lactate dehydrogenase leakage (59%), elevated reactive oxygen species (2.5-fold), depolarized mitochondrial membrane potential as confirmed by JC-1 monomers and arrested cell cycle at G2/M phase. Further, apoptotic and necrotic analysis revealed significant changes followed by DNA damage. To overcome these toxicological effects, PC was pretreated for 2 h followed by CTN exposure for 24 h. Pretreatment with PC resulted in significant increase in cell viability (84.5%), restored membrane integrity, reactive oxygen species level were maintained and cell cycle phases were normal. PC significantly up-regulated the activity of detoxification enzymes: heme oxygenase 1 (HO-1), glutathione transferase, glutathione peroxidase, superoxide dismutase and quinone reductase. Nrf2 translocation into the nucleus was also observed by immunocytochemistry analysis. These data demonstrate the protective effect of PC against CTN-induced oxidative stress in HepG2 cells and up-regulated the activity of detoxification enzyme levels through Keap1/Nrf2 signaling pathway.

**GRAPHICAL ABSTRACT:**
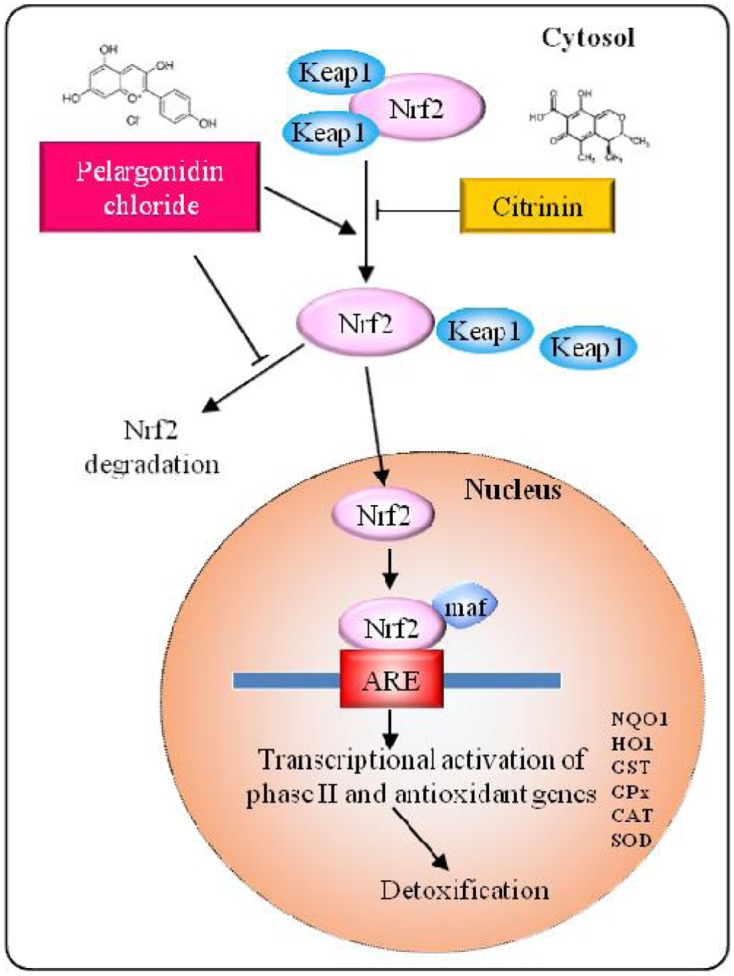
Schematic model of the Keap1/Nrf2 signaling pathway. Pelargonidin chloride dissociates Nrf2 from Keap1 resulting in translocation of Nrf2 into the nucleus, where it binds to ARE and activates cytoprotective genes.

## Introduction

Citrinin (CTN) is a fungal secondary metabolite first isolated from *Penicillium citrinum Thom* (Hetherington and Raistrick, 1931) and produced by many strains of *Penicillium*, *Aspergillus*, and *Monascus* (El-Banna et al., 1987; Blanc et al., 1995). CTN is a naturally occurring contaminant in food and feeds, and is classified as a group III carcinogen by The International Agency for Research on Cancer (International Agency for Research on Cancer [IARC], 1986). It has been implicated in human diseases such as “yellow rice” disease in Japan and Balkan Endemic Nephropathy (BEN) in some parts of southeastern Europe (Vrabcheva et al., 2000). CTN has been reported to be nephrotoxic and hepatotoxic in *in vitro* and *in vivo* (Ribeiro et al., 1997). CTN is known to affect electron transport system by altering the mitochondrial membrane in liver and kidney *in vivo* (Chagas et al., 1992). The other deleterious effects observed are, fetotoxic, embryocidal, and mildly teratogenicity (Reddy et al., 1982). At cellular level, CTN cytotoxicity is observed in a number of cell lines where its role in apoptosis and in activation of caspases, signaling pathways have been well established (Yu et al., 2006; Chan, 2007; Chang et al., 2009; Chen and Chan, 2009).

Anthocyanins are a subgroup of flavonoids responsible for imparting blue, purple and red color to many leaves, flowers, and fruits. They are water-soluble compounds present in berries, grapes, apples, red radish (Giusti and Wrolstad, 2003). Anthocyanins rich foods possess high free radical scavenging and antioxidant activity. Anthocyanins are known to have numerous health benefits and play a major role in the prevention of neuronal and cardiovascular diseases, cancer and diabetes among others (He and Giusti, 2010). Anthocyanins naturally occur as glycosides of flavylium (2-phenylbenzopyrylium) salts, and aglycones forms are called anthocyanidins. The six major anthocyanidins commonly found are: cyanidin, delphinidin, petunidin, peonidin, pelargonidin, and malvidin (Castaneda-Ovando et al., 2009). Pelargonidin (PEL) along with its glucoside form pelargonidin-3-glucoside (P3G) is known to be present in red radishes, strawberries, grapes, raspberry, mulberries and other plants, vegetables and fruits. PEL and P3G (Pelargonidin 3-*O* glucoside) have been reported to have antioxidant (Noda et al., 2002; Wang et al., 2010), anti-inflammatory (Hämäläinen et al., 2007; Lee and Bae, 2016; Min et al., 2016), antithrombotic activity (Ku et al., 2016), and antidiabetic activities (Roy et al., 2008). Pelargonidin possesses cytoprotective (Samadder et al., 2016) and antigenotoxic properties (Abraham et al., 2007), it is shown to activate AhR-CYP1A1 signaling pathway (Kamenickova et al., 2013), and plays a role in improving memory in Alzheimer’s disease *in vivo* (Roghani et al., 2010; Sohanaki et al., 2016) and also exhibits potential preventive effects toward atherosclerosis (Son et al., 2014).

Several cytoprotective genes of detoxifying and antioxidative enzymes in the xenobiotic detoxification and antioxidative response pathway are induced on exposure to electrophilic and oxidative stress. Nrf2 (nuclear factor erythroid 2-related factor 2) has been shown to mediate the cellular responses by binding to antioxidant/electrophile-responsive element (ARE/EpRE). Recent studies have reported the induction of Nrf2 by several antioxidant and chemopreventive compounds (Krajka-Kuźniak et al., 2015) where the Nrf2-Keap1 pathway has been shown to play an important role in chemoprevention Nrf2 is a strong activator of ARE regulated gene expression (Wasserman and Fahl, 1997). Keap1 (Kelch ECH associating protein 1), a cytosolic repressor protein of Nrf2 binds to Nrf2 in the cytoplasm and promotes proteasomal degradation. Keap1 acts as a sensor of electrophiles and ROS, under oxidative stress conditions, oxidants or electrophiles modify cysteine residues of Keap1 to release Nrf2 from Keap1-Cul3-Rbx1 E3 ubiquitin ligase complex (Suzuki and Yamamoto, 2015), thus activating Nrf2 and induction of cytoprotective gene expression in the nucleus. Many dietary chemopreventive compounds that have been reported to regulate or modulate Nrf2/Keap1 pathway are curcumin (Balogun et al., 2003), sulforaphane (Zhou et al., 2014), epigallocatechin gallate (Na et al., 2008), quercetin (Tanigawa et al., 2007; Granado-Serrano et al., 2012), resveratrol (Kode et al., 2008), ferulic acid (Catino et al., 2016), rosmarinic acid (Fetoni et al., 2015) etc., these compounds either function as inducers of phase I or phase II detoxification enzymes such as glutathione transferase (GST) and NAD(P)H:quinone oxidoreductase (NQO1) or via activation of HO-1 and Nrf2 antioxidant defense system.

Recent studies have reported the ability of anthocyanins to induce xenobiotic detoxification enzymes, Kumar et al. (2014), have described the differences in their ability to induce phases I and II enzymes. In the present study, we have investigated the effect of CTN on Keap1/Nrf2 pathway and the role of PC in activation of the transcription factor Nrf2 and its target genes NQO1, HO-1, GST in ameliorating CTN-induced toxicity in Human hepatocellular carcinoma (HepG2) cell line. Furthermore the ability of PC to prevent ROS levels and apoptosis, mitochondrial damage induced by CTN was also studied.

## Materials and Methods

### Chemicals

Citrinin, pelargonidin chloride, 3-(4,5-dimethylthiazol-2-yl)-2,5-diphenyl-tetrazolium bromide (MTT), 2′,7′-Dichlorofluorescin diacetate (DCFH_2_-DA), dimethyl sulfoxide (DMSO), minimum modified Eagle’s medium (MEM), hoechst 33342, propidium iodide (PI), ethidium bromide, dichlorophenol indophenol, flavin adenine dinucleotide, dicumarol, protease inhibitor cocktail, fluoroshield, RIPA buffer were purchased from Sigma–Aldrich, St. Louis, MO, United States. JC-1 (5,5′,6,6′-tetrachloro-1,1′,3,3′-tetraethyl benzimidazolyl carbocyanine iodide) and fetal bovine serum (Hyclone) was from Invitrogen, United States.

### Cell Culture and Treatments

HepG2 (Human liver hepatocellular carcinoma) cell line was supplied by National Centre for Cell Sciences, Pune, India. Cells were cultured in MEM supplemented with 10% FBS and 1% antibiotic-antimycotic solution in a humidified incubator at 37°C and 5% CO_2_. All treatments were performed in serum free media. Cell treatment procedure is as follows, HepG2 cells were exposed to CTN at 25, 50, 75, 100, and 150 μM concentrations for 24 h and pelargonidin chloride (PC) at 50 and 100 μM. The cells were preincubated with PC (50 and 100 μM) for 2 h before exposure to CTN for 24 h.

### MTT Assay for Cell Viability

MTT assay was performed as previously described (Babu et al., 2017). Cells were seeded in 24-well plates and treated as mentioned above. Twenty four hours after the treatment period, MTT solution (0.5 mg/ml) in serum free medium was added to each microwell and incubated for 2 h at 37°C. After the incubation, the MTT reagent was removed and replaced with DMSO to dissolve the formed formazan crystals. The absorbance was measured with a Plate Chameleon-Multi-technology plate reader, (HIDEX, Finland) at 570 nm and the results were expressed as percentage of control.

### Lactic Dehydrogenase Release Assay

Lactic dehydrogenase release (LDH) leakage assay was performed using Lactic acid dehydrogenase based *In vitro* toxicology assay kit, (TOX-7, Sigma–Aldrich) according to manufacturer’s instructions.

### Observation of Morphological Changes

HepG2 cells were seeded in 25 cm^2^ flasks (1 × 10^6^ cells) and treated. The cell morphology was observed under phase contrast microscope (Olympus, Tokyo, Japan) and photographed with Cat Cam 200 camera equipped with Scope photo 3.0 software.

### Assessment of Apoptosis and Necrosis

HepG2 cells were seeded on glass cover slips and grown in MEM medium with FBS for 48 h. After treatment protocol, cells were washed once with PBS and stained with acridine orange (AO, 50 μg/ml) a green fluorescent dye and ethidium bromide (EtBr, 100 μg/ml) a red fluorescent dye for 15 min at 37°C in dark. Cells were observed under Axio Imager A2 fluorescence microscope (Carl Zeiss Microscopy GmbH, Germany) and images captured with AxioVision software.

### Estimation of Intracellular Reactive Oxygen Species (ROS)

Intracellular ROS levels were determined using dichlorofluorescin diacetate (DCFH_2_-DA), as previously described by Wang and Joseph (1999) and Babu et al. (2017). After the incubation period, cells were washed with cold PBS and loaded with 10 μM DCFH_2_-DA for 30 min at 37°C, after 30 min the cells were washed to remove extracellular DCF–DA. Fluorescence signals were measured using a fluorescence micro-plate reader (Infinite M200 pro; Tecan, Grodig, Austria) at 485 nm excitation and 535 nm emission wavelength. The results were reported as the relative percent of DCF-fluorescence. For fluorescence imaging, cells were incubated with DCFDA dye and its uptake by cells was analyzed by Axio Imager A2 fluorescence microscope (Carl Zeiss Microscopy GmbH, Germany).

### Mitochondrial Membrane Potential (Δ_Ψm_) Assay Using JC-1

The mitochondrial membrane potential (Δ_Ψm_) (MMP) of intact cells was assessed by the lipophilic probe JC-1. For fluorescence ratio detection, cells were grown in 96 well plates for 48 h and treated as described before. After incubation, cells were washed once with cold PBS and stained with JC-1 dye (5 μM) for 20 min in dark at 37°C in a CO_2_ incubator. The supernatant was aspirated; cells were washed twice with PBS and 100 μl fresh PBS was added. Fluorescence intensity was measured for J-aggregates (red fluorescence) at 535 nm excitation and 595 nm emission wavelength, and JC-1 monomers (green fluorescence) excitation and emission wavelength was at 485/535 nm, respectively. The cells were analyzed in a fluorescent plate reader (Infinite M200 pro; Tecan, Grodig, Austria). The results are expressed as the ratio of fluorescent intensity of J-aggregates to monomers.

For fluorescence imaging, cells were grown in 22 mm cover slips and after treatment period, cells were stained with 2.5 μM JC-1 for 15 min at 37°C in dark. Following staining, the cover slips were washed with cold PBS and mounted onto glass microscopic slides with Fluoroshield mounting medium (Sigma–Aldrich) and examined immediately. Images were acquired using Axio Imager A2 fluorescence microscope (Carl Zeiss Microscopy GmbH, Germany).

### Cell Cycle Analysis

Cell cycle analysis was carried out as previously described (Babu et al., 2017). HepG2 cells were grown in 25 cm^2^ flasks and treated as mentioned before. After the 24 h treatment period, cells were harvested by trypsinization, centrifuged (1000 *g*, 5 min, 4°C). The cell pellet were washed with ice cold PBS and fixed with chilled 70% ethanol (Riccardi and Nicoletti, 2006). Cells were centrifuged to remove the supernatant and gently resuspended in PBS and PI staining solution was added. Cells were analyzed by Flow cytometry at a flow rate of 10,000 events per second per sample on a FACSVerse^TM^ (Becton Dickinson, United States) with FACSuite^TM^, data were analyzed using Flow Jo software.

### Single Cell Gel Electrophoresis (SCGE) Assay

To detect cellular DNA damage, a single-cell gel electrophoresis (SCGE) assay was performed according to Singh et al. (1988) with slight modifications. HepG2 cells were treated as described previously and after incubation period, treated cells and control cells were scraped, counted and adjusted to 2 × 10^4^ cells/ml in PBS. Agarose pre-coated slides were prepared with 0.75% normal melting agarose and allowed to solidify at 4°C for 5 min. 0.4 ml of cell suspension was mixed with 1.2 ml of low melting agarose (0.75%) at a volume ratio of 1:3 and rapidly pipetted on agarose-covered surface of a pre-coated microscope slide. After the gel solidified, the slides were submerged in the lysis solution (2.5 M NaCl, 100 mM EDTA, 10 mM Tris-HCl, 1% Triton X-100, 10% DMSO and 0.1% SDS, pH = 10) for 1.5 h at 4°C. The slides were transferred to an electrophoresis tank with alkaline buffer (1 mM EDTA and 300 mM NaOH, pH > 13) and allowed to stand for 20 min to allow for unwinding of DNA. The alkaline electrophoresis was carried out at 25 V, 300 mA for 20 min. The slides were washed three times with neutralization buffer (0.4 M Tris-HCl, pH 7.5) and stained with 10 μg/ml ethidium bromide. Slides were examined for comet images with a fluorescence microscope (Olympus, Japan) equipped with Cool SNAP Pro color digital camera. At least 50 comet images from each slide was examined to check DNA damage, and the comet images were analyzed by CaspLab-Comet Assay Software Project, (version 1.2) to determine the olive tail moment (OTM). The results were expressed as percent olive tail movement.

### NAD(P)H:Quinone Oxidoreductase 1 (NQO1) Assay

NAD(P)H:quinone oxidoreductase 1 activity was measured as described by Jiang et al. (2003). After treatments, cells were washed with cold PBS and homogenized in 25 mM Tris-HCl and 125 mM sucrose buffer for 30 s. Protein concentration was determined using Lowry et al. (1951) method. For measurement of quinone reductase activity, 2,6-dichloroindophenol (2,6-DCIP) served as substrate. To a 96-well microtitre plate, 5 μg of cell lysate and 200 μl of quinone reductase assay buffer containing 25 mM Tris-HCl (pH 7.4), 0.01% Tween 20, 60 μg/ml BSA, 5 μM flavin adenine dinucleotide (FAD), 80 μM 2,6-dichloroindophenol with or without dicumarol was added. Absorbance was then monitored at 600 nm over the course of 3 min. NQO1 activity was calculated as the change in absorbance min^-1^ μg^-1^ of total protein in the absence and presence of dicumarol (NQO1 inhibitor) (Hanlon et al., 2007).

### Antioxidant Enzyme Assays

HepG2 cells were cultured in 75 cm^2^ flasks and treated as mentioned before. Antioxidant enzyme assays were performed as previously described (Babu et al., 2017). After 24 h incubation period the cells were washed with PBS and then 0.5 ml of homogenization buffer (50 mM sodium phosphate buffer, pH 7.4 containing 2 mM EDTA and 0.1% Triton X-100) was added to each flask and incubated for 24 h in a -80°C freezer. The cells were thawed and homogenized in cold conditions; lysate was centrifuged at 12,000 *g* for 20 min at 4°C. The supernatants were collected for protein concentration determination by Lowry et al. (1951) method using bovine serum albumin as a standard. The activity of antioxidant enzymes, glutathione peroxidase (GPx) and superoxide dismutase (SOD) was measured using kits as per the manufacturer’s instructions (Cat no., RS504, SD125 Randox, Mississauga, ON, Canada).

Catalase (CAT) activity assay was performed spectrophotometrically according to Aebi (1984) by measuring the rate of hydrogen peroxide (H_2_O_2_) (10 mM) decrease at 240 nm at room temperature for 3 min. Specific activity or the rate of decomposition of H_2_O_2_ by catalase was calculated from the equation:

$$\text{Specific activity }({\text{units}}^{-1}\,\text{mg }{\text{protein}}^{-1}{\text{min}}^{-1})=\Delta {\text{A}}_{\left(\text{240 nm}\right)}/\text{min}\times 1000/43.6\text{ mg protein}$$

### Estimation of Total Glutathione

Total glutathione (GSSG + GSH) was determined using Glutathione Assay Kit (CS0260, Sigma–Aldrich) according to manufacturer’s instructions. HepG2 cells were homogenized in 5% 5-sulfosalicylic acid solution and centrifuged at 12,000 *g* for 15 min to remove protein precipitates and assayed for glutathione. The end product, 5-thio-2-nitrobenzoic acid (TNB) was measured spectrophotometrically at 412 nm and expressed as nmoles GSH per mg of protein.

### cDNA Synthesis and Quantitative Reverse-Transcription Polymerase Chain Reaction

RNA extraction was carried out using RNeasy Mini Kit (Qiagen, Hilden, Germany) according to manufacturer’s instructions. One microgram of total RNA was converted into cDNA using Superscript III First strand synthesis system (Invitrogen, Van Allen Way, Carlsbad, CA, United States) as per the manufacturer’s instructions. The quantitative real time polymerase chain reaction (qPCR) was performed using the iTaq^TM^ Universal SYBR Green Supermix (Bio-Rad, Hercules, CA, United States) on the Bio-Rad CFX96^TM^ Real-Time PCR system (Bio-Rad, Hercules, CA, United States). All target primers were predesigned KiCqStart primers (Sigma–Aldrich) and are listed in **Table 1**. The reaction mixture was then subjected to thermal profile of denaturation at 95°C for 30 s, followed by amplification and quantification in 40 cycles at 95°C for 10 s and 60°C for 30 s. The calculated threshold cycle was normalized to the value of the internal control β-actin expression and mean fold change was quantified using the 2^-ΔΔ*C*_T_^ method (Livak and Schmittgen, 2001).

**Table 1:** Primers sequences used for real-time PCR reactions.

Gene	Sequence	NCBI accession Number	Amplicon size (bp)
*Nrf2*	F- CGTTTGTAGATGACA ATGAGG	NM_001145412	122
	R- AGAAGTTTCAGGTGACTGAG		
*HO-1*	F- CAACAAAGTGCAAGATTCTG	NM_002133	150
	R- TGCATTCACATGGCATAAAG		
*NQO1*	F- AGTATCCACAATAGCTGACG	NM_000903	134
	R- TTTGTGGGTCTGTAGAAATG		
*Keap1*	F- GCACAACTGTATCTATGCTG	NM_012289	167
	R- CTCCAAGGACGTAGATTCTC		
*GSTA1*	F- AGGTATAGCAGATTTGGGTG	NM_145740	129
	R- AAGACTTTTTCAAAGGCAGG		
*ACTB*	F- GATCAAGATCATTGCTCCTC	NM_001101	191
	R- TTGTCAAGAAAGGGTGTAAC		
*CAT*	F- AGAGAAATCCTCAGACACATC	NM_001752	161
	R- CAGCTTGAAAGTATGTGATCC		
*GPx1*	F- CTACTTATCGAGAATGTGGC	NM_000581	162
	R- CAGAATCTCTTCGTTCTTGG		
*SOD1*	F- GAGCAGAAGGAAAGTAATGG	NM_000454	136
	R- GATTAAAGTGAGGACCTGC		


### Cell Extracts, Subcellular Fractionation and Immunoblots

After incubation with pelargonidin chloride and CTN for the indicated time points, the cells were washed twice with PBS and lysed using ice-old RIPA lysis buffer and 1x protease inhibitor cocktail. The obtained cell homogenates were centrifuged at 12,000 *g* for 30 min at 4°C, the supernatants was collected followed by protein estimation by Lowry et al. (1951) method. Nuclear extraction was done according to Joung et al. (2007), cells were obtained by centrifugation and resuspended in ice-cold isotonic buffer A consisting 10 mM HEPES (pH 7.9), 1.5 mM MgCl_2_, 10 mM KCl, 0.5 mM dithiothreitol (DTT), and 0.2 mM phenylmethylsulfonyl fluoride (PMSF) and allowed to stand on ice bath for 10 min. Nuclear fraction was collected by centrifugation and again the nuclear fraction was lysed for 20 min in ice cold buffer B consisting 20 mM HEPES (pH 7.9), 0.2 mM EDTA, 420 mM NaCl, 1.5 mM MgCl_2_, 20% glycerol, 0.5 mM DTT and 0.1 mM PMSF. The lysed nuclei suspension was centrifuged again to collect the final nuclear extract. The protein concentration was determined by Lowry et al. (1951) method. Equal amount of protein (50 μg) was subjected to SDS-PAGE and following electrophoresis, the gel was transferred to a polyvinylidenedifluoride (PVDF, Millipore, Bedford, MA, United States) membrane. The membranes were kept for blocking overnight at 4°C with 5% (w/v) skim milk powder and 0.05% Tween-20 in Tris-buffered saline (10 mM Tris-HCl pH 7.5, 150 mM NaCl). After blocking, the membranes were incubated with primary Nrf2 (sc-722), HO-1 (H105 sc-10789), GAPDH (sc-25778), p53 (C-11 sc-55476), Bcl2 (C-2 sc-7382), Bax (6D150 sc-70408), Cytochrome-C (sc-13156), NQO1 (C-19 sc-16464), Keap1 (E-20 sc-15246), CAT (sc-34280), SOD (sc-8637), and GPx (sc-22146) antibodies (1:1000) for 2 h. The membranes were washed 4–5 times with TBST for 5 min and secondary incubation was carried out with polyclonal goat anti-rabbit, rabbit anti-goat and goat anti-mouse (Dako, Glostrup, Denmark) horseradish peroxidase-conjugated secondary antibodies (1:5000) respectively. The membranes were again washed five times at 5 min intervals to remove excess secondary antibody. The immunoreactivity was detected using the kit chemiluminescent peroxidase substrate (CPS-160, Sigma–Aldrich) according to manufacturer’s instructions. The signals were captured on a CL-XPosure Film (Thermo Scientific, Waltham, MA, United States) and the intensity of protein bands were quantified using NIH Image J software.

### Laser Confocal Microscopy

HepG2 cells were seeded on to 18 mm cover slips (ethanol and UV sterilized) in six well plates for 2–3 days and was treated as described before. Cells were fixed in 4% paraformaldehyde for 15 min at room temperature, permeabilized with 0.25% Triton X-100 in PBS (pH 7.4). After blocking with 1% bovine serum albumin (BSA) in PBS for 30 min, they were incubated with Nrf2 (sc-722, Santa Cruz Biotechnology) at 1:500 dilution in PBS containing 1% BSA for overnight at 4°C. Nrf2 was detected with the secondary antibody conjugated with FITC (F0382; Sigma–Aldrich) and washed with PBS. The cells were counterstained with hoechst 33342 and mounted in Fluoroshield mounting medium onto a glass microscopic slide. Immunofluorescence analysis was performed with a laser scanning confocal imaging system coupled to a FV1000 Confocal Microscope (Olympus, Japan). Cells were viewed with a 63× objective lens (Numerical aperture 1.35), and images were captured with FluoView software (Olympus, Japan).

### Statistical Analysis

The values are expressed as the mean ± standard deviation and repeated three times. The experimental data was analyzed using one-way analysis of variance (ANOVA) and Tukey’s *post hoc* test by SPSS statistical software (version 20.0; SPSS, Inc., Chicago, IL, United States). Differences were considered significant when the *p*-values were <0.05.

## Results

### Effect of PC on CTN-Induced Cytotoxicity in HepG2 Cells

**FIGURE 1:**
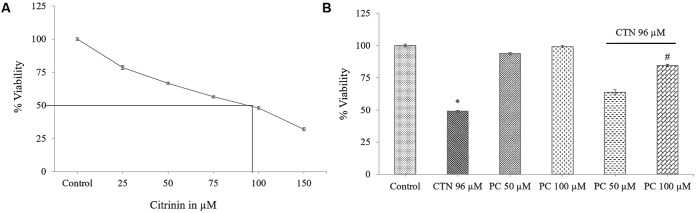
Effect of CTN on cell viability as measured by MTT assay. **(A)** Dose-response curve of CTN. **(B)** HepG2 cells were pretreated with PC (50 and 100 μM) for 2 h followed by with or without CTN (96 μM) treatment for 24 h. Data are expressed as the mean ± SD (*n* = 3). ^∗^*p <* 0.05 vs. control cells, ^#^*p* < 0.05 vs. CTN treated cells.

Cytotoxicity was determined colorimetrically by MTT dye method. HepG2 cells were seeded in 24 well plates and CTN was treated in a dose dependent manner (25, 50, 75, 100, and 150 μM) for 24 h. After incubation, a dose dependent decrease in cell viability was observed and the IC_50_ value was found to be of 96.16 μM (**Figure 1A**). Based on the dose response, effective concentration of CTN was fixed at 96.16 μM. At 100 μM the cell viability decreased to 48.08% (*p* < 0.05 vs. control). No toxicity was observed when PC (50 and 100 μM) was exposed to HepG2 cells for 24 h. To test the cytoprotective efficacy of pelargonidin chloride, HepG2 cells were pretreated with PC for 2 h at concentrations 50 and 100 μM followed by CTN treatment for 24 h. As shown in **Figure 1B**, PC at 50 and 100 μM inhibited CTN-induced cytotoxicity and cell viability was maintained at 63.69 and 84.49% (*p* < 0.05) respectively.

**FIGURE 2:**
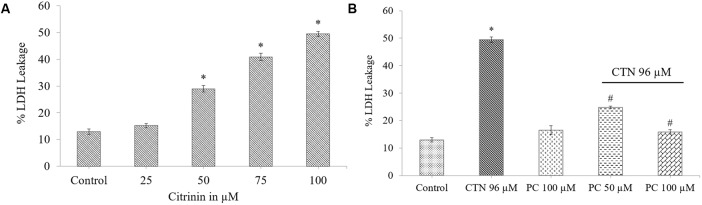
Effect of CTN on LDH leakage into the medium. **(A)** HepG2 cells after treatment with different concentrations of CTN (0–100 μM) for 24 h. **(B)** Protective effect of PC (50 and 100 μM) when pretreated for 2 h followed by with or without CTN (96 μM) treatment for 24 h. Data are expressed as the mean ± SD (*n* = 3). ^∗^*p* < 0.05 vs. control cells, ^#^*p* < 0.05 vs. CTN treated cells.

**FIGURE 3:**
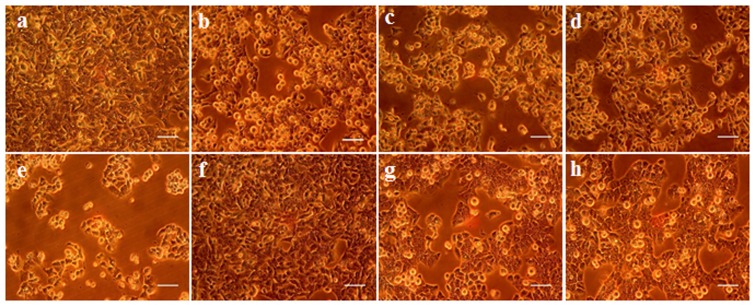
Effect of PC on the morphology of HepG2 cells, **(a)** Control, **(b–e)** CTN 25, 50, 75, and 100 μM, **(f)** PC 100 μM, **(g)** PC 50 μM + CTN 96 μM, **(h)** PC 100 μM + CTN 96 μM. HepG2 cells were treated with PC for 2 h followed by with or without CTN (96 μM) treatment for 24 h and morphological changes observed under phase-contrast microscopy. The figures shown are representative of three independent experiments. Scale bar, 100 μm.

Lactic dehydrogenase leakage into the medium is a measure of plasma membrane integrity and after 24 h a dose-dependent increase of 29, 41, and 49% LDH was observed at 50, 75, and 100 μM CTN concentrations (*p* < 0.05) (**Figure 2A**). Pretreatment of HepG2 cells with PC significantly (24.5 and 15.9%) (*p* < 0.05) protected the membrane from LDH leakage when compared to CTN treated cells (**Figure 2B**). Distinct morphological characteristics were observed when HepG2 cells were exposed to CTN in a dose dependent manner. HepG2 cells showed characteristic apoptotic features such as cell shrinkage, blebbing and rounding off when compared to control cells (**Figures 3a–e**). PC pretreatment protected the cells from CTN-induced cell damage and death (**Figures 3g,h**).

### Effect of PC on the CTN-Induced Apoptosis and/or Necrosis

**FIGURE 4:**
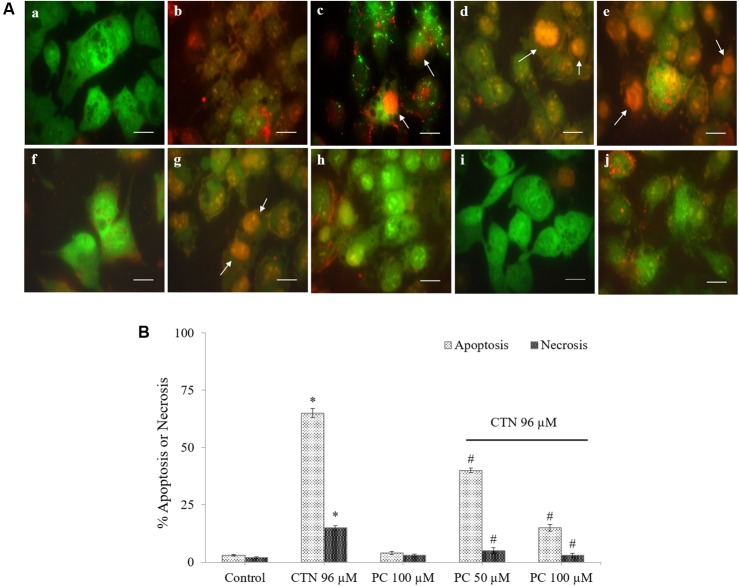
Representative photomicrographs of HepG2 cells stained with acridine orange (AO, green) and ethidium bromide (EtBr, red) fluorescent dyes, **(A)** Vital cells (green), early apoptotic (bright green to yellow), late apoptotic (orange) and necrotic cells (red). **(a)** Control, **(b–e)** CTN 25, 50, 75, and 100 μM, **(f)** Control, **(g)** CTN 96 μM, **(h)** PC 100 μM, **(i)** PC 50 μM + CTN 96 μM, **(j)** PC 100 μM + CTN 96 μM. **(B)** The percentage of apoptotic and necrotic cells after staining with AO and EtBr. Data are expressed as the mean ± SD. ^∗^*p* < 0.05 vs. control cells, ^#^*p* < 0.05 vs. CTN treated cells. Scale bar, 20 μm.

The percentage of apoptotic and necrotic cells were analyzed by fluorescence microscopy using the dyes acridine orange (AO) and ethidium bromide (EtBr). CTN treatment significantly caused changes in cell morphology and dye uptake in a dose dependent manner (**Figures 4Aa–e**). Early apoptotic cells are seen as bright green to yellow, while late apoptotic and necrotic cells show orange and red characteristics. PC alone did not induce apoptosis or necrosis at the concentrations tested (**Figure 4Ag**). Pretreatment with 50 and 100 μM of PC resulted in cytoprotection and significantly reduced apoptotic and necrotic cells (**Figures 4Ai,j**). The percentage of apoptotic and necrotic cells after staining with AO and EtBr is shown in **Figure 4B**.

### Effect of PC on the CTN-Induced Production of ROS

**FIGURE 5:**
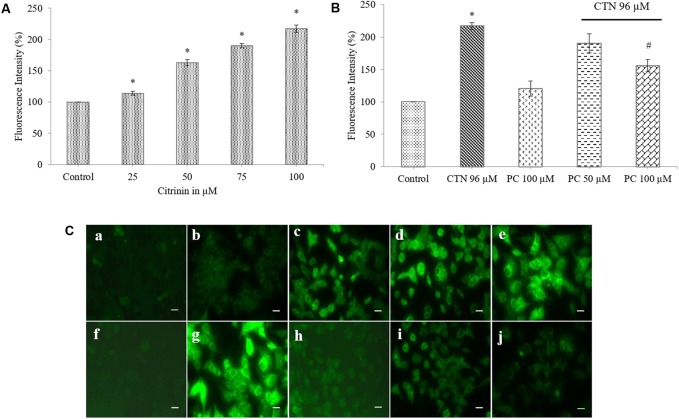
Effect of PC on CTN-induced ROS in HepG2 cells. **(A)** Cells were treated with CTN for 24 h and then intracellular ROS levels were detected. ROS levels were found to be increased compared to control cells. **(B)** PC pretreatment for 2 h followed by with or without CTN (96 μM) treatment for 24 h markedly decreased intracellular ROS. **(C)** Fluorescence images of HepG2 cells stained with H_2_DCF-DA, ROS accumulation leads to oxidation of the dye resulting in increased DCF fluorescence (green color) **(a)** Control, **(b–e)** CTN 25, 50, 75, and 100 μM, **(f)** Control, **(g)** CTN 96 μM, **(h)** PC 100 μM, **(i)** PC 50 μM + CTN 96 μM, **(j)** PC 100 μM + CTN 96 μM. Scale bar, 50 μm. Data are expressed as the mean ± SD (*n* = 3). ^∗^*p* < 0.05 vs. control cells, ^#^*p* < 0.05 vs. CTN treated cells.

The ability of CTN to oxidize H_2_DCF-DA was compared by measuring the fluorescence increase by a fluorescence plate reader and microscopy. The intracellular ROS accumulation was measured as percentage DCF florescence. H_2_DCF-DA was used here to determine whether changes in the intracellular level of ROS correlated with the apoptosis induced by CTN. CTN (25–100 μM) treatments increased ROS accumulation in a dose-dependent manner in HepG2 cells compared to control cells (**Figure 5A**, *p* < 0.05). At 100 μM, ∼2.2-fold increase in ROS accumulation was observed. In the presence of PC (50 and 100 μM) ROS generation decreased significantly, PC pretreatment (100 μM) effectively inhibited ROS with a 1.3-fold decrease (*p* < 0.05) relative to CTN treated cells (**Figure 5B**). The differences in ROS production among the CTN doses as determined by fluorescence plate reader were confirmed by using fluorescence microscopy (**Figure 5C**). No DCF fluorescence was detected in control and PC treated cells. The addition of CTN to HepG2 cells resulted in a large increase in DCF fluorescence (**Figures 5Ca–e**). However, cells pretreated with PC showed a weaker DCF fluorescence signal compared to CTN treated cells.

### Effect of PC on CTN-Induced Loss of MMP (Δ_Ψm_)

**FIGURE 6:**
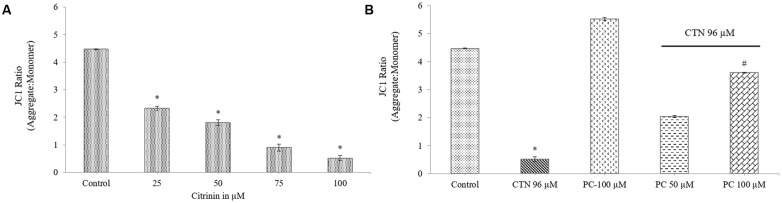
The mitochondrial membrane potential is expressed as JC-1 fluorescence ratio in terms of red fluorescence to green fluorescence. **(A)** Effect of CTN on loss of MMP in HepG2 cells. **(B)** PC pretreatment for 2 h prevented loss of MMP by CTN (96 μM) treatment. Data are expressed as the mean ± SD (*n* = 3). ^∗^*p* < 0.05 vs. control cells, ^#^*p* < 0.05 vs. CTN treated cells.

**FIGURE 7:**
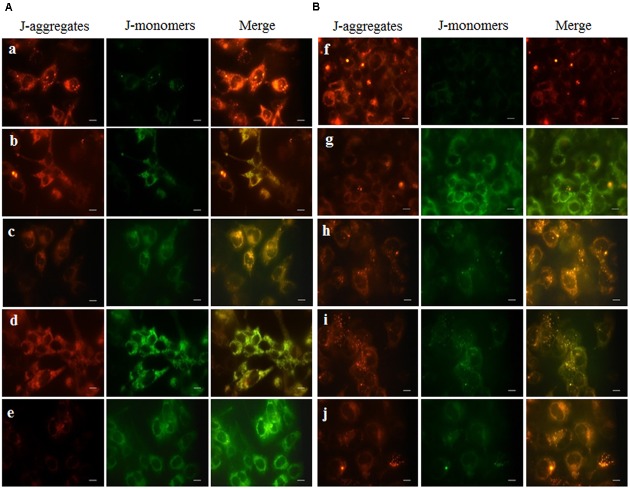
Fluorescent microscopic images of HepG2 cells stained with JC-1 dye after CTN treatment. Red color indicates healthy cells with high MMP and green color indicates low MMP. **(A)** Effect of CTN on loss of MMP in HepG2 cells **(a)** Control, **(b–e)** CTN 25, 50, 75, and 100 μM. **(B)** PC pretreatment for 2 h prevented loss of MMP by CTN (96 μM) treatment **(f)** Control, **(g)** CTN 96 μM, **(h)** PC 100 μM, **(i)** PC 50 μM + CTN 96 μM, **(j)** PC 100 μM + CTN 96 μM. Scale bar, 50 μm.

The ability of mitochondria to maintain their trans-membrane potential was assayed by JC-1 dye. JC-1 is a ratiometric probe and the Δ_Ψm_ was monitored by determining the relative amounts of dual emissions from mitochondrial J-aggregates (red fluorescence) to JC-1 monomers (green fluorescence). Control cells and PC alone had no effect on the Δ_Ψm_. CTN significantly depolarized the Δ_Ψm_ as manifested by declined red/green fluorescent ratio of JC-1 compared to control cells (**Figure 6A**) (*p* < 0.05). This effect was significantly counteracted by PC pretreatment (**Figure 6B**). HepG2 cells were incubated with JC-1 (**Figures 7A,B**), which exists either as a green-fluorescent monomer at depolarized membrane potentials or as an orange/red-fluorescent J-aggregate at hyperpolarized membrane potentials. As shown in **Figure 7Aa**, in control cells the intensity of red fluorescence was detected which appeared as rod-like structures which showed that mitochondria are hyperpolarized and JC-1 is converted to aggregates. When CTN was exposed to HepG2 cells, there was dose dependent decrease in red fluorescence intensity and a very small amount of J-aggregates were detected at 50 and 75 μM. At 96 and 100 μM CTN, HepG2 cells exhibited complete green fluorescence indicating that all JC-1 remained in monomeric form (**Figures 7e,g**). Pretreatment with PC inhibited CTN-induced mitochondrial membrane potential loss which was evident from orange color rod shaped structures (**Figures 7i,j**). These results suggest that CTN significantly decreased the Δ_Ψm_ in HepG2 cells and further confirmed the participation of a mitochondria-related mechanism in the apoptosis.

### Effect of PC on CTN-Induced Arrest of Cell Cycle Phases

**FIGURE 8:**
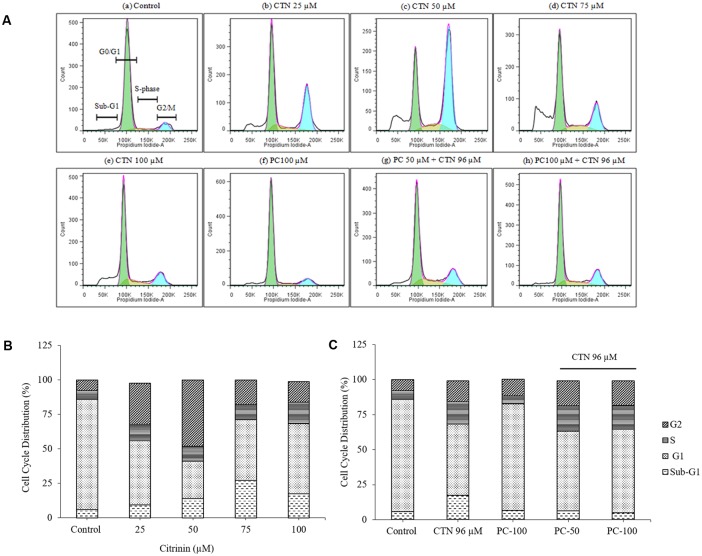
Cell cycle analysis using propidium iodide (PI) staining and flow cytometry. **(A)** Flow cytometry histogram of HepG2 cells after CTN treatment and compared with pretreatment with PC. **(B)** Cell cycle phase distribution (%) of 24 h CTN treated HepG2 cells showing G2/M phase arrest and sub-G1 phase. **(C)** Cell cycle phase distribution (%) of PC pretreated (2 h) cells showing protection after 24 h CTN exposure.

HepG2 cells when treated with CTN showed important change in the percentages of cells in different cell cycle phases. After 24 h, a significant decrease in number of cells from all tested CTN concentrations was observed in G0/G1 phase (**Figures 8A,B**). 50 μM CTN concentration showed the least amount with 26.97%. HepG2 cells treated with CTN demonstrated cell-cycle arrest, with an increased number of cells in the G2/M phase after 24 h of treatment in a dose dependent manner. CTN treatment caused accumulation of cells by 29.92, 48.15, 17.94, and 14.96% (25, 50, 75, and 100 μM) respectively, in G2/M phase. The highest DNA content in G2/M phase was observed at 50 μM. The percentage of S-phase cells was found to be 15.56% in CTN treated cells (100 μM) compared to 6.26% in control cells. A hypodiploid cell population was observed in sub-G1 phase indicating the presence of apoptotic nuclei. The proportion of cells in sub-G1 phase increased dose dependently upon CTN treatment (25–100 μM) (**Figures 8A,B**) with 27.33% being highest at 75 μM. No effect on cell cycle phases was observed with PC (50 and 100 μM) after 24 h of treatment in HepG2 cells (**Figures 8A,C**). Pretreatment with PC inhibited apoptosis and the percentage of sub-G1 phase cells reduced (**Figure 8C**) markedly. Thus, PC at both the concentrations was effective in inhibiting CTN-induced G2/M phase arrest.

### Effect of PC Pretreatment on SCGE Assay

**FIGURE 9:**
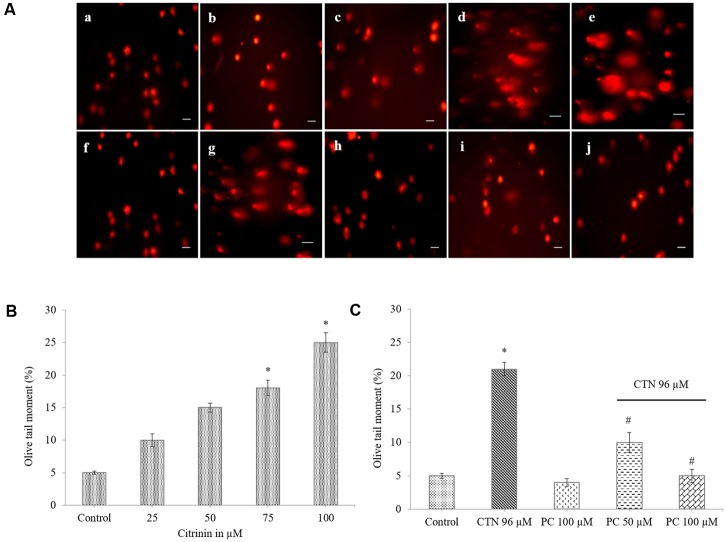
Effect of PC on DNA damage induced by CTN. **(A)** Comet images in different treatment groups **(a)** Control, **(b–e)** CTN 25, 50, 75, and 100 μM, **(f)** Control, **(g)** CTN 96 μM, **(h)** PC 100 μM, **(i)** PC 50 μM + CTN 96 μM, **(j)** PC 100 μM + CTN 96 μM. **(B)** Effect of CTN on DNA damage in HepG2 cells. **(C)** Protective effect of PC on olive tail moment when pretreated for 2 h followed by with or without CTN (96 μM) treatment for 24 h. Scale bar, 50 μm.

DNA damage caused by CTN was assessed in HepG2 cells by alkaline Comet assay/SCGE assay. Treatment of HepG2 cells with varying concentrations of CTN (25–100 μM) for 24 h resulted in a maximum induction of DNA damage at 100 μM (**Figures 9A,B**). The mean tail length, tail intensity and olive tail moment (OTM) at 25 and 50 μM was very less and non-significant but at higher CTN (100 μM) concentration, the OTM was visible and found to be significant (*p* < 0.05) compared to control cells. DNA damaged was inhibited by PC pretreatment and the tail length was also decreased which is used as a parameter for detecting DNA damage (**Figures 9A,C**).

### PC Ameliorates CTN Induced Oxidative Stress and Restores GSH Levels

**FIGURE 10:**
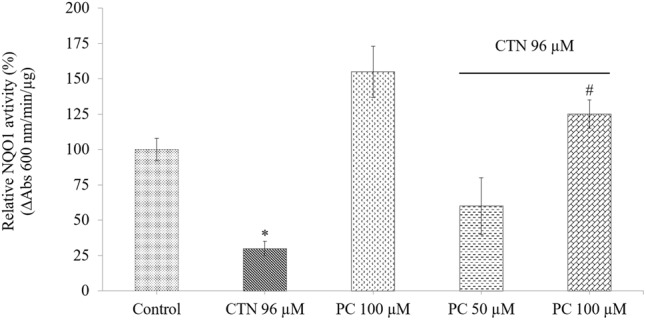
Measurement of NQO1 activity in HepG2 cells. Data are expressed as the mean ± SD (*n* = 3). ^∗^*p* < 0.05 vs. control cells, ^#^*p* < 0.05 vs. CTN treated cells.

The quinone reductase enzymatic activity (NQO1) of HepG2 cells were induced by 155% upon PC treatment (*p* < 0.05) after 24 h. While pretreatment with PC for 2 h followed by CTN treatment showed strong detoxifying enzyme activity (**Figure 10**). The cytoprotective properties of PC in counteracting CTN-induced deleterious effects was studied with respect to changes in antioxidant enzymes levels of the following CAT, SOD, and GPx enzymes, respectively. CTN exposure significantly decreased the antioxidant activity of CAT, SOD, and GPx enzymes (*p* < 0.05) (**Table 2**). The inhibitory effects of CTN on CAT, SOD, and GPx activity were significantly attenuated by PC pretreatment. Glutathione (GSH) is a tri-peptide (γ-glutamylcysteinylglycine) antioxidant, total glutathione (GSH, reduced + GSSG, oxidized) levels were measured in cells treated with CTN (96 μM) and pretreated with PC. There was significant GSH depletion in CTN treated cells. As shown in **Table 2**, PC pretreatment increased GSH levels (*p* < 0.05) compared to CTN alone treated cells.

**Table 2:** Effect of PC pre-treatment on antioxidant status in HepG2 cells against CTN-induced toxicity.

Treatment groups	Catalase (mM/mg protein)	SOD (U/mg protein)	GPx (μM/mg protein)	Total GSH (nmoles/mg protein)
Control	0.100 ± 0.02	6.51 ± 0.32	3.43 ± 0.02	210.75 ± 9.10
CTN 96 μM	0.038 ± 0.01^∗^	1.62 ± 0.46^∗^	0.94 ± 0.01	62.89 ± 6.90^∗^
PC 100 μM	0.083 ± 0.01	5.92 ± 0.13	3.15 ± 0.01	203.60 ± 4.34
PC 50 μM + CTN 96 μM	0.043 ± 0.03^#^	3.82 ± 0.61	1.53 ± 0.01	109.11 ± 2.43^#^
PC 100 μM + CTN 96 μM	0.075 ± 0.02^#^	5.12 ± 0.27^#^	2.46 ± 0.02	176.49 ± 5.21^#^


*Data are expressed as the mean ± standard deviation from three independent experiments, each performed in triplicate. ^∗^*p <* 0.05 vs. control cells; ^#^*p < 0.05* vs. CTN treated cells.*

### Effect of PC on the mRNA Expression Level of Phases I and II Cytoprotective Genes

**FIGURE 11:**
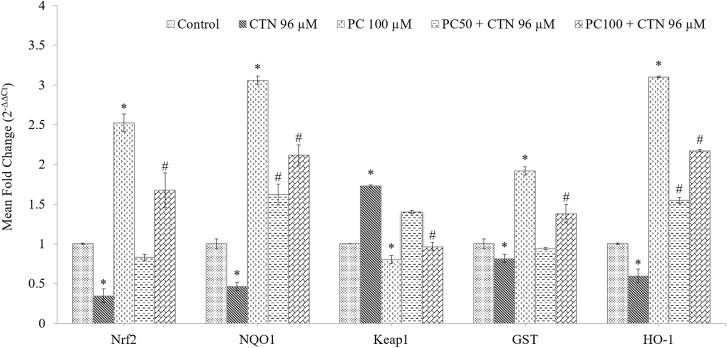
Effect of PC on mRNA expression levels of Nrf2, NQO1, Keap1, GST and HO-1 in HepG2 cells. mRNA was analyzed by quantitative real-time PCR; normalized gene expression levels are given as the mean fold change. Data are expressed as the mean ± SD (*n* = 3). ^∗^*p* < 0.05 vs. control cells, ^#^*p* < 0.05 vs. CTN treated cells.

**FIGURE 12:**
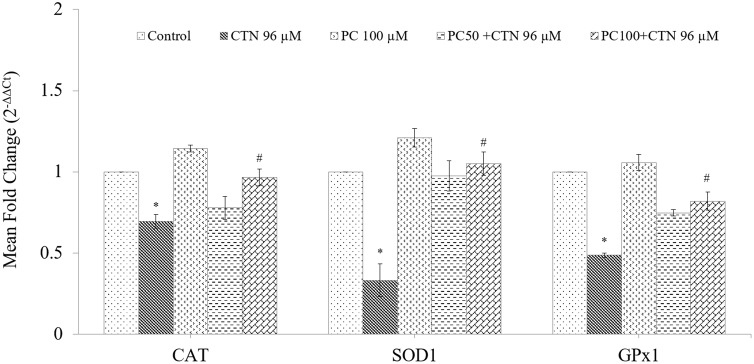
Effect of PC on Nrf2-mediated mRNA expression levels of antioxidant enzymes, CAT, SOD1, and GPx1. mRNA was analyzed by quantitative real-time PCR; normalized gene expression levels are given as the mean fold change. Data are expressed as the mean ± SD (*n* = 3). ^∗^*p* < 0.05 vs. control cells, ^#^*p* < 0.05 vs. CTN treated cells.

The mRNA expression of cytoprotective genes was measured by quantitative PCR. As shown in **Figure 11**, the mRNA expression levels of Nrf2 were decreased by CTN treatment followed by increase in Keap1 expression compared to control group. However, the mRNA expression levels of Nrf2 were up-regulated by 2.5-fold in PC (100 μM) alone treated cells (*p* < 0.05) compared to control group. PC pretreatment resulted in activation of Nrf2 gene as evidenced by significant increase in its two transcriptional targets HO-1 and NQO1. The level of mRNA transcripts of GST was also significantly increased by PC treatment. The effect of PC on antioxidant gene expression of CAT, SOD1, and GPx1 increased after pretreatment with PC (**Figure 12**).

### Effect of PC on the Protein Expression Level of Phase II and Antioxidant Enzymes

**FIGURE 13:**
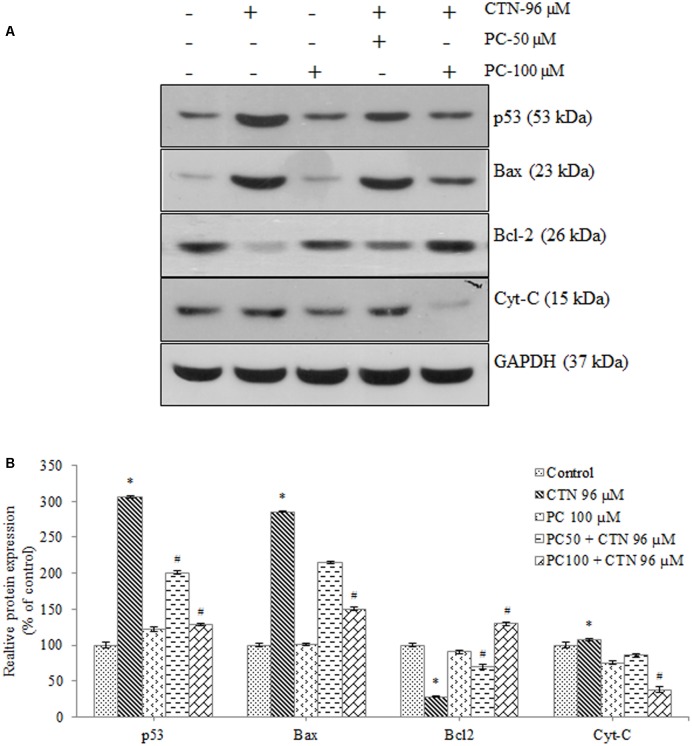
Effect of PC on the expression of apoptotic markers, **(A)** Expression of p53, Bax, Bcl2 and Cyt-C analyzed by western blotting. **(B)** Densitometry analysis of the intensity of the protein bands. Data are expressed as the mean ± SD (*n* = 3). ^∗^*p* < 0.05 vs. control cells, ^#^*p* < 0.05 vs. CTN treated cells.

**FIGURE 14:**
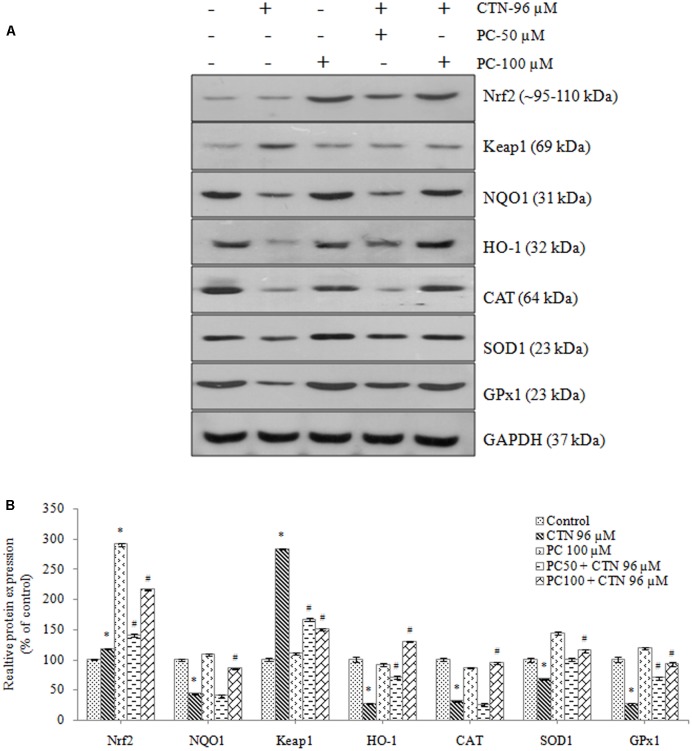
Western blot analysis of phase II detoxification proteins and antioxidant protein markers, **(A)** Expression of Nrf2, Keap1, NQO1, HO-1, CAT, SOD1, and GPx1 upon pretreatment with PC for 2 h followed by with or without CTN (96 μM) treatment for 24 h. **(B)** Densitometry analysis of the intensity of the protein bands. Data are expressed as the mean ± SD (*n* = 3). ^∗^*p* < 0.05 vs. control cells, ^#^*p* < 0.05 vs. CTN treated cells.

The expression of Bcl-2 was noticeably decreased as shown in **Figure 13**, while Bax activity in CTN treated group was significantly higher than that of the control group (*p* < 0.05). Pretreatment with PC suppressed the activities of Bax triggered by excessive oxidative stress. These findings suggest that Bcl-2 family of proteins is involved in the apoptosis induced by CTN. As shown in **Figure 12**, following treatment with CTN (96 μM), there is an increase in cytochrome-C (Cyt-C) protein expression, while PC pretreated cells down-regulate CTN-induced mitochondrial damage and release of Cyt-C. The changes in Nrf2 protein expression in response to CTN exposure are shown in **Figure 14**. Pretreatment with PC caused up-regulation of nuclear Nrf2 further supporting transcriptional activation of Nrf2 in response to CTN exposure in HepG2 cell line. The downstream antioxidant proteins of the Nrf2 signaling pathway were found to be down-regulated by CTN treatment and PC pretreatment elevated Nrf2 protein expression. The expression of CAT, SOD1, and GPx1 also increased after pretreatment with PC (Supplementary Data Sheet 1).

### Immunocytochemistry

**FIGURE 15:**
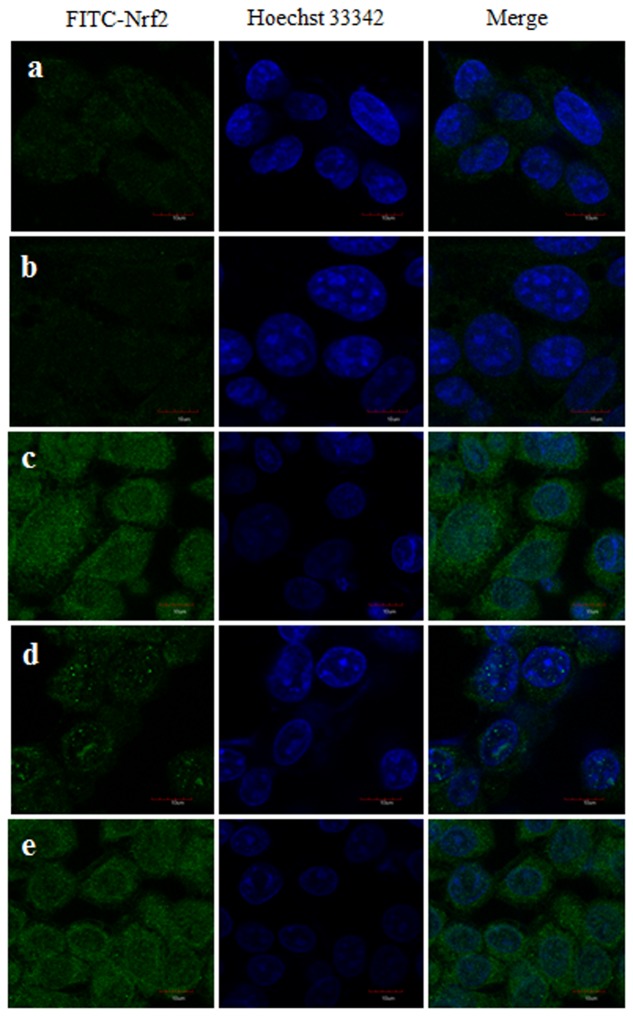
Nrf2 nuclear localization in HepG2 cells was examined by confocal microscopy after pretreatment with PC for 2 h followed by with or without CTN (96 μM) treatment for 24 h **(a)** Control, **(b)** CTN 96 μM, **(c)** PC 100 μM, **(d)** PC 50 μM + CTN 96 μM, **(e)** PC 100 μM + CTN 96 μM. Green signals represent nuclear distribution of Nrf2 while nuclei were stained with Hoechst 33342 (blue signal) as assessed by secondary antibody conjugated with FITC staining in laser scanning confocal microscope. Scale bar, 10 μm.

Expression of the Nrf2 protein was assessed by confocal microscopy. There was no nuclear translocation when HepG2 cells were exposed to CTN for 24 h. Upon PC pretreatment Nrf2 protein was visible in nucleus where Nrf2 translocation occurred, and undetectable levels of nuclear Nrf2 in the absence of PC treatment (**Figure 15**). Nuclei were stained with Hoechst 33342 to monitor the localization of the Nrf2 induction and to aid in assessing the increase in Nrf2.

## Discussion

The human intestine, liver and kidney are organs rich in drug-metabolizing enzymes, which interact with drugs and food constituents. The xenobiotic-metabolizing enzymes, NQO1, GST, and HO-1 are the most important and are generally distributed enzymes responsible for more than two thirds of metabolic processes with known mechanisms. Phase II detoxification genes or oxidative stress response genes are, involved in cellular defense against ROS, ROS-induced cell cycle arrest, DNA damage responses, and maintenance of cellular redox state. A better understanding of the mechanisms by which CTN causes toxicity is essential for effective management of CTN-induced diseases.

Hormesis is a dose response phenomenon characterized by low dose stimulation and a high dose inhibition (Calabrese et al., 2007). HepG2 cells show dose responses (Calabrese, 2005) and the activity of PC is an example of physiological conditioning hormesis in which pretreatment with PC reduces the toxicity of CTN. Most of the anthocyanins and athocyanidins are water soluble and PC is one among them which makes it effective in cytoprotection upon CTN-induced cellular stress and this natural phytocompound can be used against hepatotoxicity. There is now considerable evidence of how phytocompounds sulforaphane, curcumin, isothiocyanates and resveratrol exert protective effect upon various stress and oxidative stimuli. The hormetic mechanism of action may account for its role in health benefits of these phytocompounds (Noyan-Ashraf et al., 2006). Some of the hormetic phytochemicals that are known to induce expression of cytoprotective phase II proteins through Keap1/Nrf2/ARE pathway are sulforaphane (Zhou et al., 2014), tert-butylhydroquinone (Prochaska et al., 1992), curcumin (Dinkova-Kostova and Talalay, 1999), ferulic acid (Ma et al., 2010), resveratrol (Kode et al., 2008) etc.

Nrf2 is bound to Keap1 and inhibited under basal conditions but later undergoes Cul3-Rbx1 ubiquitination system for proteasomal degradation. Upon induction by xenobiotic/electrophiles/oxidant factors, Nrf2 is released from Keap1 for phosphorylation of Nrf2, enters nucleus, and activates Nrf2 pathway (Qin and Hou, 2016). Disruption of the actin cytoskeleton is thought to promote nuclear translocation of Nrf2 reporter protein. Keap1 is a chemical sensor responsible for Nrf2 activation during oxidative and electrophilic stress. The actin cytoskeleton provides scaffolding that is essential for the function of Keap1 (Kang et al., 2004). Based on these previous reports and our data, anthocyanins and phytochemicals can induce the expression of several oxidative stress-related molecules (Kumar et al., 2014; Qin and Hou, 2016), we hypothesized that Nrf2 might play an important role in this induction. It has been reported that the expression of several antioxidative proteins that have antioxidant response element in the promoter region can be induced by Nrf2 stimulation (Suzuki and Yamamoto, 2015). Our present study showed that the effects of CTN-induced ROS generation, cell cycle arrest and modulation of Nrf2 and HO-1 gene expression and activation of xenobiotic-metabolizing enzymes are altered by PC in response to toxicological insult.

HepG2 cells when treated with CTN undergo toxic insult with concomitant increase in oxidative stress. PC pretreatment tends to activate the detoxification signaling pathway genes responsible for protection which are under the control of genes called vitagenes. Vitagenes are a set of genes which are responsible for maintaining cellular homeostasis during stress (Calabrese et al., 2009, 2010b). The transcription factor Nrf2 upon binding to ARE element in nucleus encodes phase II proteins and downstream antioxidant enzymes called vitagenes (HO-1, Hsp70, thioredoxin, thioredoxin reductase).

Recent studies have reported that anthocyanins are able to induce the activation of phase II enzymes. In a study by Shih et al. (2007) anthocyanins such as cyanidin, kuromanin, cyanidin, and malvidin showed higher efficacy in their antioxidant capacity and induction of phase II enzymes. Other major anthocyanins which are known to have protective effects are cyanidin 3-O-β-d-glucoside on ochratoxin A (OTA) toxicity (Russo et al., 2005), cyanidin-3-O-β glucopyranoside on OTA and aflatoxin B1 toxicity (Guerra et al., 2005) and cyanidin-3-*O-*glucoside (C3G) on antiatherogenic effects.

As the liver is one of the main target organs of CTN, HepG2 cell line is often used as a model for toxicity studies. Recent studies have demonstrated CTN’s ability to induce apoptosis and increase intracellular ROS (Yu et al., 2006; Chan, 2007). Administration of CTN exerted clear toxicity at concentrations above 50 μM and complete decrease in cell viability at 150 μM (**Figure 1A**). In order to overcome the cytotoxicity, PC was pretreated for 2 h followed by co-incubation with CTN for 24 h. PC pretreatment effectively inhibited CTN’s toxicity and, cell viability was maintained to 84% which was significant when compared to CTN only treated cells (**Figure 1B**). Necrosis and apoptosis are two different modes of cell death that differ in morphology, mechanism and occurrence. CTN caused membrane disruption, metabolic collapse, cell swelling and rupture leading to cell death, which is a typical feature of necrosis. Apoptosis is characterized by cell shrinkage, chromatin condensation and DNA cleavage (Majno and Joris, 1995). The apoptotic and necrotic events results were further confirmed by LDH assay, morphological changes, double staining by AO/EtBr (**Figures 2**–**4**). PC is a strong antioxidant and its ability to scavenge free radicals generated by CTN might be one of reason for its protective effect.

Previous studies involving CTN have shown to generate intracellular ROS (Chan, 2007; Gayathri et al., 2015) and induce oxidative stress. In our study, ROS was measured based on the oxidation of H_2_DCF-DA to fluorescent DCF. H_2_DCF-DA is a cell permeable non-fluorescent dye used as an intracellular probe for oxidative stress (Wang and Joseph, 1999). The fluorescence signal in this assay is indicative of accumulated intracellular ROS mediated by CTN. The rate of H_2_DCF-DA oxidation increased ∼2.2-fold after CTN exposure to HepG2 cells (**Figure 5C**). The results are in accordance with the previous data on the induction of oxidative stress by CTN (Chan, 2007; Gayathri et al., 2015). Excessive production of ROS may be one of the mechanism by which CTN have induced DNA damage as evidenced by the protein expression of its apoptotic targets p53, Bax and Bcl2 (**Figure 13**). The increased ROS levels, apoptotic markers and DNA damage were ameliorated by PC pretreatment which can be correlated with previous studies by Shih et al. (2007) where anthocyanins suppressed H_2_O_2_ induced apoptosis in clone 9 cell line. The results obtained by the SCGE assay are in accordance with those obtained in a previous study (Gayathri et al., 2015) where the induction of unscheduled DNA synthesis was evaluated in HepG2 cell line. The findings described in this study show that PC plays a role in lowering ROS formation in HepG2 cells. Mitochondrial depolarization can be observed by a decrease in the red/green fluorescence intensity ratio as indicated by a fluorescence emission shift from red (∼590 nm) to green (∼529 nm) (Salvioli et al., 1997). JC-1 is less toxic to cells which can be solubilized easily and appropriate pKa and fluorescence characteristics which make it convenient for detection by epifluorescence microscopy (Smiley et al., 1991). CTN treated HepG2 cells show a gradual decrease in electrochemical gradient and fluorescent intensity (**Figure 6**). J-aggregate formation is largely membrane potential dependent, a reduction in the plasma membrane potential should also lead to a reduction in J-aggregate formation (Smiley et al., 1991) which was observed in the case of CTN where JC-1 remained as monomers (**Figure 7**). CTN affects mitochondrial permeability transition at the cellular level as well as dysfunction of mitochondria resulting in the loss of mitochondrial membrane potential and release of Cyt-C from mitochondria to cytosol (Chagas et al., 1992; Ribeiro et al., 1997; Da Lozzo et al., 1998). Our results demonstrated that pretreatment with 100 μM PC effectively prevented Cyt-C release from the mitochondria to the cytosol and other apoptotic factors Bax and p53 were also inhibited (**Figure 13**). Based on the literature, the result of oxidative stress is thought to be DNA damage, which in turn leads to p53 activation during certain cellular stress responses (Kruse and Gu, 2009). Flow cytometry analysis is widely used for evaluating cell death, apoptosis and cell cycle parameters. Previous studies have reported the arrest of cell cycle in the G2/M phase (Chang et al., 2010) and accumulation of Sub G1 phase apoptotic cells upon CTN treatment. The present study showed that CTN increased the percentage of cells in the sub-G1 phase and arrested the cell cycle phase at the G2/M phase (**Figure 8**). The data presented is consistent with the findings of the previous studies where a similar effect was observed (Yu et al., 2006; Hsu et al., 2012). Also, the percentage of hypodiploid cells in sub-G1 phase of flow cytometry correlated with that of SCGE assay where DNA damage is reported (**Figure 9**). Antioxidant enzyme activity of CAT, SOD, GPx were also decreased upon CTN treatment and were maintained to their basal levels by PC pretreatment (**Table 2**). To evaluate cellular redox state, total glutathione levels were assessed which is responsible for protecting cells from oxidative stress and ROS accumulation. Our results showed that CTN decreased GSH levels which were overcome upon pretreatment with PC to maintain antioxidant defense against CTN-induced hepatotoxicity.

Further studies were carried out to confirm the activity of PC by quantitative real time-PCR where mRNA levels of Nrf2-regulated enzymes including NQO1, GST, and HO-1 were analyzed in response to CTN treatment. Our study showed that, CTN alters the expression of phase II and downstream antioxidant genes in HepG2 cells and down-regulated major genes, such as HO-1, GST, and NQO1.

Pelargonidin chloride pretreatment modulates Nrf2 expression and exerts its antioxidant effects by upregulating the expression of Nrf2 (**Figure 11**). The Nrf2 protein in the cytoplasm is translocated into the nucleus and Nrf2 activity is significantly increased. It has been reported that Nrf2 binds to the promoter region of ARE in the nucleus at the 5′-flanking region and promotes the activation of several phase II detoxification and antioxidant gene expression. Based on the earlier studies by Itoh et al. (1997), he found that activation of gene expression is through the binding of Nrf2 to the ARE region with high affinity with a small Maf protein. Activation of Keap1/Nrf2 signaling pathway also leads to induction of antioxidant enzymes, such as GPx, SOD, glutathione reductase (GR) and GSH all of which have the ability to scavenge xenobiotics (Lu, 2009). PC pretreatment was able to significantly increase the mRNA and protein expression of the phases I and II detoxification enzymes (**Figures 12**, **14**). Our gene and protein expression data suggest that PC may mediate disruption of Keap1 dimerization and trigger the release of Nrf2 in HepG2. Many studies have also been carried to show the Keap1 complex disruption and Nrf2 release. Keap1 complex disruption may occur through an, (a) Erk- and p38-independent mechanism *in vivo* (Zipper and Mulcahy, 2002), (b) by Nrf2 inducing ability of protein kinase C (Huang et al., 2000) and phosphatidylinositol 3-kinase (Kang et al., 2000; Lee et al., 2001), (c) from thiol modification of cysteine residues of Keap1 resulting in conformational change (Zipper and Mulcahy, 2002), (d) ability of ARE-inducing agents and their reactivity with sulfhydryl groups (Dinkova-Kostova et al., 2001). Overall, modification of Keap1 cysteine moiety may play a role in the release of Nrf2. Nrf2 nuclear translocation involves not only Keap1 oxidation but Nrf2 phosphorylation. Translocation of cytosolic Nrf2 to nucleus represents the prerequisite event of receptor activation. This was also confirmed by immunocytochemical analysis demonstrating high levels of nuclear Nrf2 in the cells treated with PC (**Figure 15**).

HO-1 is a member of heat shock protein (Hsp) family of heat shock transcription factor also referred to as Hsp32 (Calabrese et al., 2009). Lin et al. (2007) have reported that HO-1 is highly inducible in response to various stimuli, including oxidative stress and speculated that nuclear localization of HO-1 protein may serve to up-regulate genes that promote cytoprotection against oxidative stress. Based on our gene and protein expression studies it is evident that PC induced up-regulation of HO-1 protein via Keap1/Nrf2 pathway with subsequent release of cytoprotective proteins, such as GST, NQO1. HO-1 is an early responder against various stress, electrophiles and oxidants (Calabrese et al., 2010a). HO-1 induction has shown to have neuroprotective effects in both *in vitro* and *in vivo* studies (Dattilo et al., 2015) and it effects are due to biliverdin production in an heme degradation pathway resulting in cytoprotective properties (Mancuso and Barone, 2009). A study by Palozza et al. (2006) describes another mechanism by which human lung cancer cells (Mv1Lu) react to oxidative stress by repressing HO-1 expression and a knock down of Bach1 gene restored HO-1 expression.

The plasma membrane redox system (PMRS) plays an important role in the response of cells to membrane-associated oxidative stress by transferring electrons from reduced nicotinamide adenine dinucleotide phosphate [NAD(P)H] and ascorbate to extracellular free radicals/oxidants (Calabrese et al., 2010a). One of the enzymes of the PMRS, NQO1 mediates hormetic responses of HepG2 cells to CTN-induced increase in oxidative stress. NQO1 is mainly present in liver and can be induced by phytochemicals or protective agents (De Long et al., 1986). NQO1 is a ubiquitous flavoprotein found in most eukaryotes mostly in cytosol, it is responsible for converting reactive quinones to relatively stable hydroquinones (Jiang et al., 2003). NQO1 is a well-defined Nrf2-ARE regulated enzyme that protects cells against naturally occurring xenobiotics-containing quinone moieties by converting exogenous quinones into hydroquinones through a two-electron reduction pathway (Buettner, 1993; Pink et al., 2000). Since CTN is a quinone (Poupko et al., 1997), our study showed that it inhibited NQO1 synthesis (**Figure 10**). PC pretreatment counteracted these effects through NQO1-mediated production of the hydroquinone that can be easily conjugated and removed from the cell (Ross et al., 1985; Pink et al., 2000).

Glutathione transferases are cytosolic isoenzymes and phase II detoxification enzymes and a large number of human alpha-class GSTs have been identified with A1–A4 (Sheehan et al., 2001). Our qPCR results show that GSTA1 gene expression in CTN treated group was less than compared to control cells. PC pretreatment showed a marked increase in GSTA1 gene expression and neutralized the toxic effects of CTN. GSTs catalyze the conjugation of GSH by attaching the sulfhydryl group of GSH to the electrophilic compounds in order to inactivate the electrophile and make it more water soluble (Tew, 1994; Armstrong, 1997). In Nrf2/Keap1 pathway, Nrf2 coordinately regulates the expression of GSTs and NQO1 (Itoh et al., 2010) and loss of transcription factor Nrf2 results in reduction in the expression of GSTs (Chanas et al., 2002). These data conclude that activation of Keap1/Nrf2 pathway is essential to detoxify the xenobiotics.

Apart from CTN, mycotoxins such as, OTA (Cavin et al., 2007; Boesch-Saadatmandi et al., 2008, 2009; Ramyaa and Padma, 2013) and Zearalenone (Wu et al., 2014; Long et al., 2016) have shown to inhibit Nrf2/ARE signaling pathway in *in vitro* and *in vivo* studies. Taken together, the results indicate that PC is involved in the activation of Nrf2/ARE-dependent gene expression and further activate detoxification enzymes. Further studies are required to establish the protective effect of PC against CTN-induced toxicity in animal model.

## Conclusion

The present study has shown the adverse effects of CTN in HepG2 cells leading to decrease in cell viability, membrane disintegration resulting in apoptosis and cell death. CTN at increasing concentrations induced ROS accumulation, mitochondrial dysfunction and arrested cell cycle at G2/M phase. PC pretreatment protected the cells dose-dependently and decreased CTN-induced cytotoxicity, and has cytoprotective effects on CTN-induced cellular oxidative stress and apoptosis. PC up-regulated the expression of stress response genes (Nrf2, NQO1, HO-1, GST) and increased intracellular antioxidant capability thus protecting the HepG2 cells against CTN insult. Overall, the results of the present showed that PC was effective in ameliorating the effects of CTN-induced hepatotoxicity. Further studies on the mechanism of phase I and Phase II detoxification system is needed for the better understanding of mycotoxins induced hepatotoxicity.

## Author Contributions

Contribution to the conception or design of the work; GS, TA, and NI. Acquisition and analysis; GS, TA, and NI. Interpretation of data for the work; GS, TA, and NI. Drafting the work; GS, TA, NI, FK, and NG. Revised it critically for important intellectual content; GS, TA, NI, FK, and NG. Final approval of the version to be published; GS, TA, NI, FK, and NG. Agreement to be accountable for all aspects of the work in ensuring that questions related to the accuracy or integrity of any part of the work are appropriately investigated and resolved. GS, TA, NI, FK, and NG.

## Conflict of Interest Statement

The authors declare that the research was conducted in the absence of any commercial or financial relationships that could be construed as a potential conflict of interest.
