# Supplementary material for: Pelargonidin Modulates Keap1/Nrf2 Pathway Gene Expression and Ameliorates Citrinin-Induced Oxidative Stress in HepG2 Cells
Source: Front Pharmacol. 2017 Nov 27;8:868. doi: 10.3389/fphar.2017.00868 (PMC5711834; doi:10.3389/fphar.2017.00868)
Supplement: Supplementary file 1 [file Data_Sheet_1.pdf]

Supplementary:

Pelargonidin modulates Keap1/Nrf2 pathway gene expression and ameliorates citrinin-induced oxidative stress in HepG2 cells

Sharath Babu G R[1], Anand T[1]*, Ilaiyaraja N[1], Farhath Khanum[1], Gopalan N[2]

[1]Biochemistry and Nano Sciences Division, Defence Food Research Laboratory, Mysuru-570011, Karnataka, India

[2]Food Biotechnology Division, Defence Food Research Laboratory, Mysuru-570011, Karnataka, India

***Corresponding author**

Biochemistry and Nano Sciences Division,
Defence Food Research Laboratory,
Mysuru-570011, Karnataka, India
E-mail address: anand@dfrl.drdo.in
Tel.: 0821-2579487; Fax: 0821-2473468

Figure S1 and S2 Original Western blots

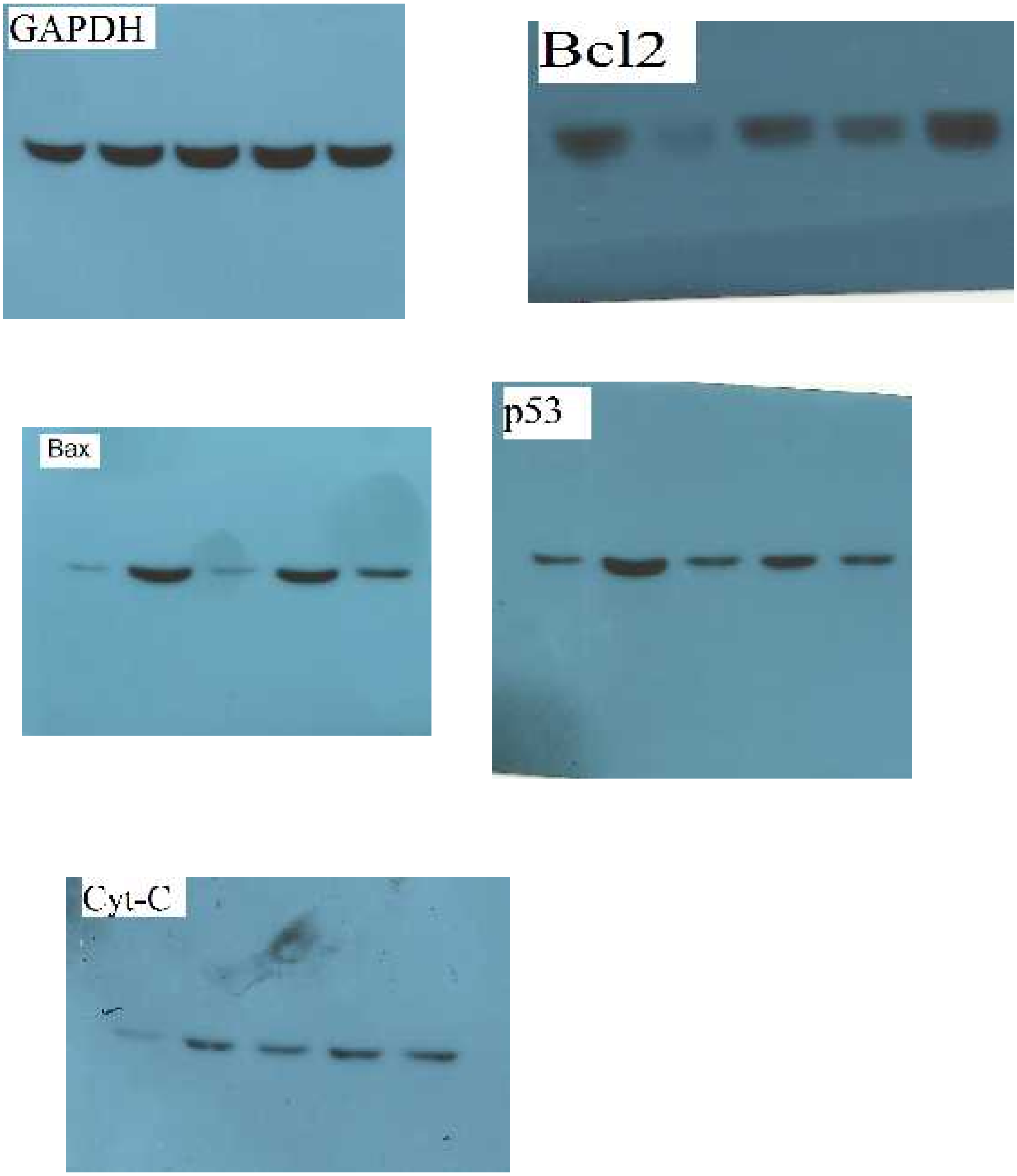

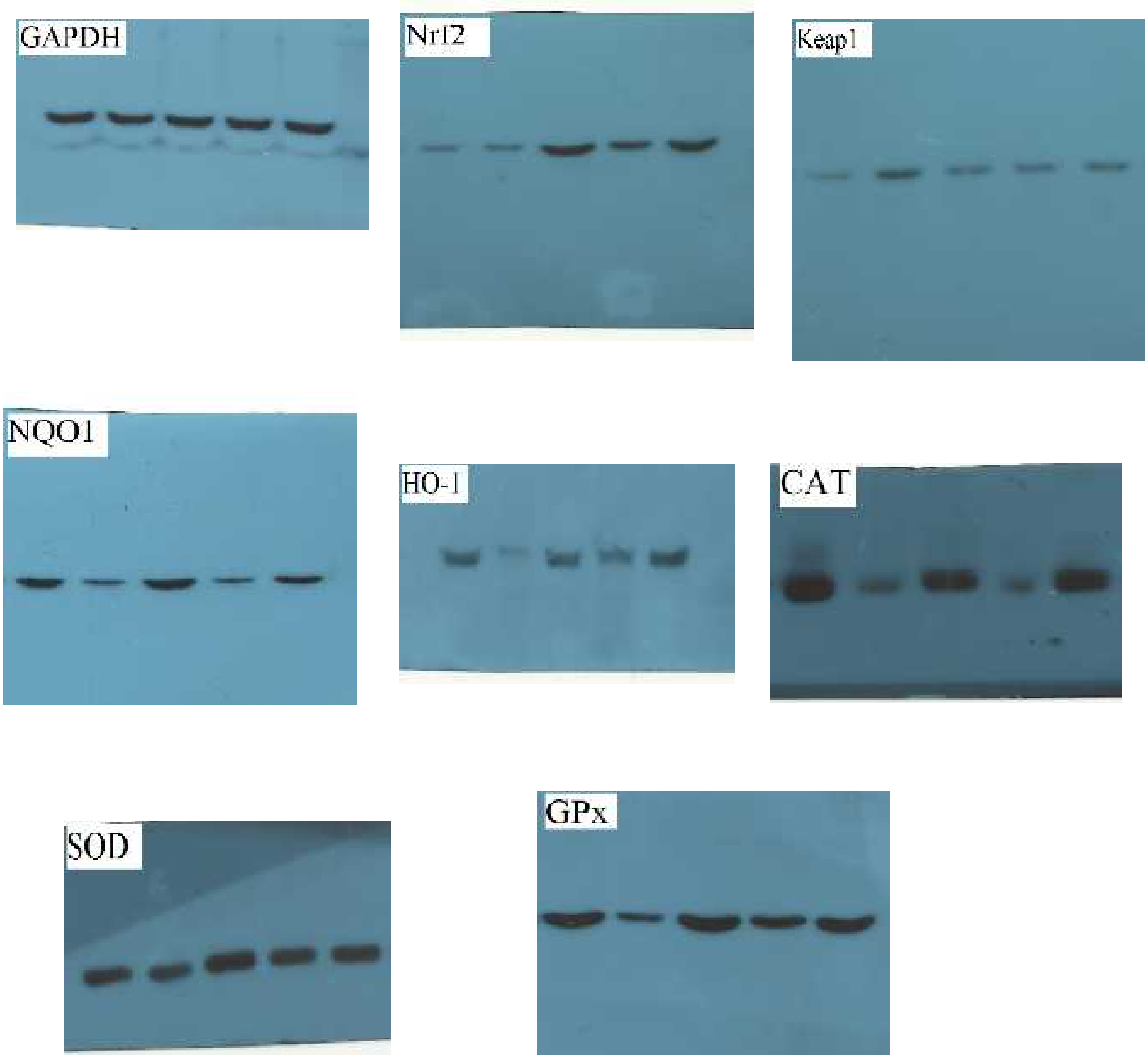
GAPDH
Nrf2
Keap1
NQO1
HO-1
CAT
SOD
GPx
